# Life in the lumen: The multivesicular endosome

**DOI:** 10.1111/tra.12715

**Published:** 2019-12-19

**Authors:** Jean Gruenberg

**Affiliations:** ^1^ Biochemistry Department University of Geneva Geneva Switzerland

**Keywords:** ALIX, anthrax, bis(monoacylglycero)phosphate BMP, calcium store, cholesterol, enveloped virus, ESCRTs, exosome, intralumenal vesicle ILV, lipidomics, lysobisphosphatidic acid, lysosome, lysosome storage disease, multivesicular endosome, Niemann‐pick C, pathogen, penetration, toxin

## Abstract

The late endosomes/endo‐lysosomes of vertebrates contain an atypical phospholipid, lysobisphosphatidic acid (LBPA) (also termed bis[monoacylglycero]phosphate [BMP]), which is not detected elsewhere in the cell. LBPA is abundant in the membrane system present in the lumen of this compartment, including intralumenal vesicles (ILVs). In this review, the current knowledge on LBPA and LBPA‐containing membranes will be summarized, and their role in the control of endosomal cholesterol will be outlined. Some speculations will also be made on how this system may be overwhelmed in the cholesterol storage disorder Niemann‐Pick C. Then, the roles of intralumenal membranes in endo‐lysosomal dynamics and functions will be discussed in broader terms. Likewise, the mechanisms that drive the biogenesis of intralumenal membranes, including ESCRTs, will also be discussed, as well as their diverse composition and fate, including degradation in lysosomes and secretion as exosomes. This review will also discuss how intralumenal membranes are hijacked by pathogenic agents during intoxication and infection, and what is the biochemical composition and function of the intra‐endosomal lumenal milieu. Finally, this review will allude to the size limitations imposed on intralumenal vesicle functions and speculate on the possible role of LBPA as calcium chelator in the acidic calcium stores of endo‐lysosomes.

## Graphical abstract

Multivesicular late endosomes contain the atypical phospholipid LBPA, which is abundant in intralumenal membranes/vesicles (ILVs). The role of LBPA will be discussed, as well as ILV functions and mechanisms that drive ILV biogenesis in light of their diverse composition and fate. The review will also explore how ILVs are hijacked by pathogens, and discuss the biochemical composition of the lumenal milieu, the size limitations imposed on ILV functions and possible roles of LBPA as calcium chelator in acidic calcium stores.

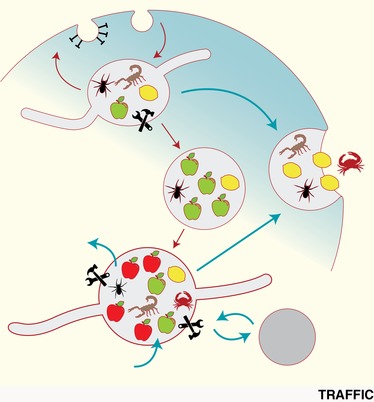

## 1 SETTING THE STAGE: THE ENDOSOMAL SYSTEM

**Figure 1:**
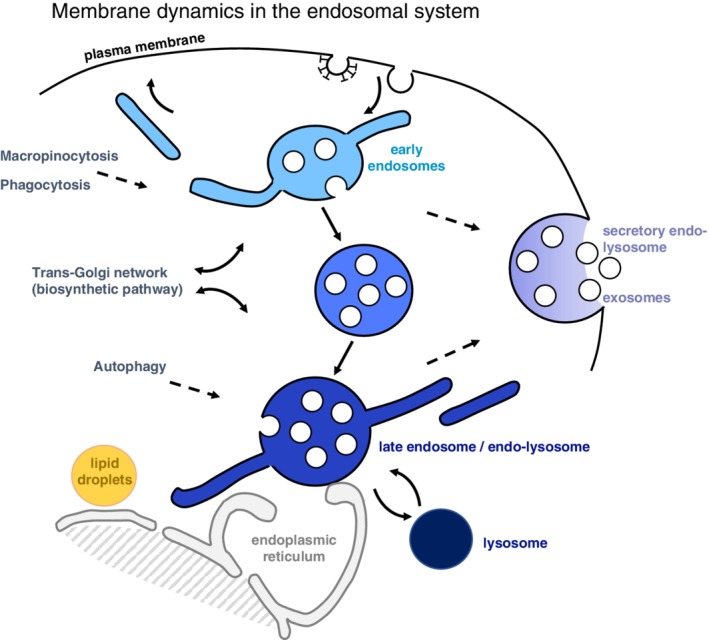
Outline of the endocytic pathway. Organization of the endosomal pathway in mammalian cells, but not in yeast or plant cells.^2^ Endocytosed components are delivered to a common early endosome, from where some proteins and lipids are recycled back to the plasma membrane, or routed by retrograde transport to the trans‐Golgi network. Molecules destined for late endosomes are sorted into ILVs forming on early endosomal membranes, giving rise to multivesicular endosomes. These detach (or mature) from early endosomes and transports cargoes toward late endosomes and lysosomes. Eventually, some ILVs are delivered to lysosomes where they are degraded together with their protein cargo. Late endosomes and lysosomes exchange membrane components and solutes, forming a transient hybrid endo‐lysosome, which is then re‐converted into secondary lysosomes, where hydrolases are stored. Endosomes and lysosomes can also undergo fusion with the plasma membrane as secretory endo‐lysosomes, and ILVs can also be released extracellularly as exosomes. The endosomal pathway also serves as an input or output for other membrane trafficking pathways, as indicated. In particular, endosomes and lysosomes also function at a crossroad with the autophagy pathway, and engage in physical contacts via membrane contact sites with other organelles, including the endoplasmic reticulum

The endosomes of eukaryotic cells are at center stage in controlling the reutilization vs degradation of membrane components, and thus regulate fundamental cellular processes in nutrient uptake, immunity, signaling, adhesion, membrane turnover and development. Components that have been endocytosed by several pathways are delivered to a common early endosome, from where some lipids and proteins, including housekeeping receptors, are recycled back to the plasma membrane (Figure 1), and others are routed by retrograde transport to the trans‐Golgi network (TGN).^1^, ^2^, ^3^ By contrast, molecules that are destined for late endosomes and lysosomes, including activated signaling receptors, are selectively sorted into lumenal invaginations, which are pinched off as free cargo‐containing intralumenal vesicles (ILVs). These multivesicular regions detach or mature from early endosomes and become multivesicular endosomes (or multivesicular bodies) that transport cargoes toward late endosomes and lysosomes.^1^, ^2^, ^4^

Late endosomes and lysosomes rapidly exchange membrane components and solutes in vivo leading to the prevailing notion that, upon fusion, they form a transient hybrid endo‐lysosome, which is then re‐converted into secondary lysosomes, where hydrolases are stored^1^, ^2^, ^5^, ^6^ (Figure 1). As a result, in this network, the net distinction between late endosomes, endo‐lysosomes and lysosomes is often blurred.^2^ Late endosomes also function at a crossroad with the autophagy pathway, which, in addition to endocytosis and TGN‐derived traffic, provides an additional entry point in the endocytic pathway for the degradation of cytoplasmic material, including organelles.^7^, ^8^, ^9^ In addition, endosomes engage in physical contacts with other organelles, including in particular the endoplasmic reticulum, via membrane contact sites that play a key role in lipid movement, calcium exchange and endosome dynamics.^10^, ^11^, ^12^, ^13^, ^14^

Endosomes and lysosomes can also acquire the capacity to fuse with the plasma membrane as secretory endo‐lysosomes—a process reminiscent of the regulated exocytosis of lysosome‐related organelles in specialized cell types.^15^, ^16^, ^17^ As a consequence, ILVs not only mediate protein and lipid transport to lysosomes for degradation, but can also be released extracellularly as exosomes, which package cellular molecules that, upon delivery to target cells, regulate a wide range of functions at a distance from the exosome‐secreting cell.^18^, ^19^, ^20^, ^21^ ILVs may also meet additional fates in specialized cell types,^22^ and contribute to the biogenesis of melanosomes in melanocytes,^23^, ^24^ or harbor MHC class II molecules loaded with peptides for presentation at the plasma membrane in antigen‐presenting cells.^25^, ^26^, ^27^ They may also undergo back‐fusion with the endosome limiting membrane^28^, ^29^, ^30^—as do exosomes after endocytosis by the target cell.^31^ Well‐integrated with the above functions, late endosomes serve as key sensing/signaling platforms that inform the cell about the cell nutrient situation.^2^, ^32^, ^33^

## 2 AN ATYPICAL LIPID WITH TWO NAMES

**Figure 2:**
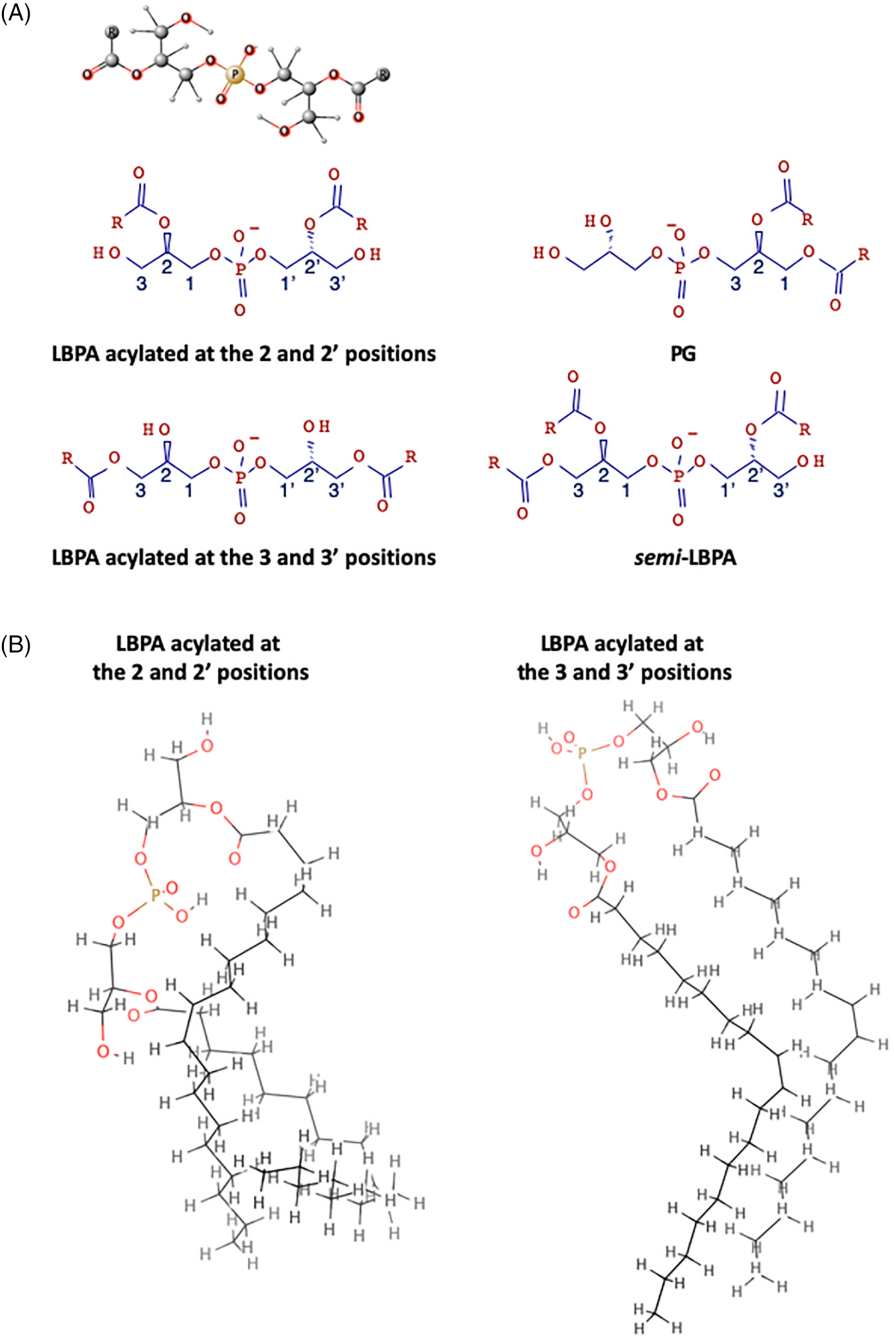
LBPA and isoforms. A, LBPA vs PG and LBPA isoforms. The ball‐and‐stick model of LBPA acylated at the 2 and 2′ positions is shown on top of the figure, above the schematic representations of the same isoform, as well as LBPA acylated at the 3 and 3′ positions, PG and *semi*‐LBPA. B, LBPA acylated at the 2 and 2′ positions vs LBPA acylated at the 3 and 3′ positions. The outlines show the atomistic description by molecular dynamics at the quantum mechanical level of two of the lowest energy conformers for both 2,2’‐LBPA and 3,3’‐LBPA^83^

Lysobisphosphatidic acid (LBPA) was discovered as a structural isomer of phosphatidyl glycerol (PG) in 1967 by Body and Gray^34^ (Figure 2), close to a decade after PG,^35^ and a century after the description of the first phospholipid (lecithin or phosphatidyl choline).^36^, ^37^ Soon after its discovery, it was found that LBPA accumulates in the lysosomal storage disease Niemann‐Pick at a time when a precise diagnosis of this lipidosis was uncertain,^38^ and later that the lipid is enriched in rat liver lysosomes.^39^, ^40^ In the early 70s, LBPA was re‐named bis(monoacylglycero)phosphate (bis[MAG]P or today BMP)^39^ —a name unfortunately easily confused with the cognate bone morphogenetic factor (≈ 17.000 citations in PubMed).

**Figure 3:**
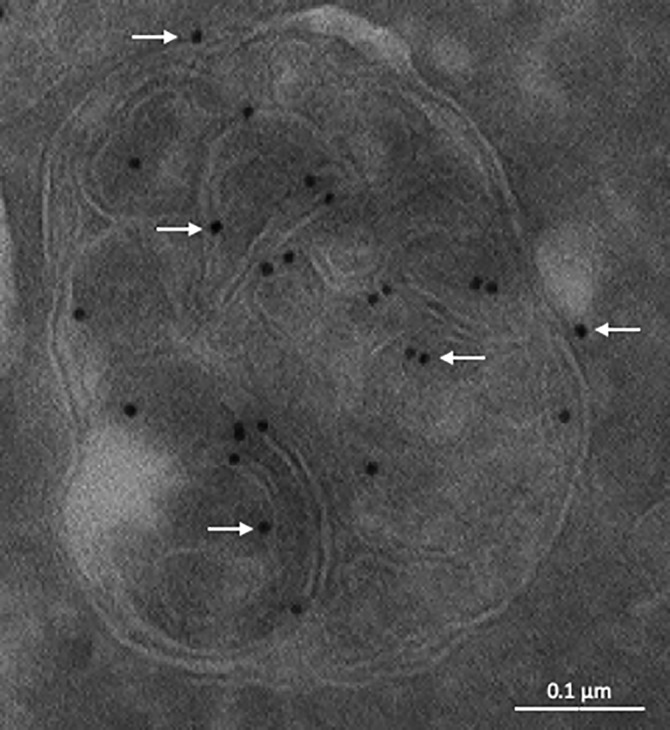
Distribution of LBPA in late endosomes illustrated by immunogold labeling of cryosections. The electron micrograph shows a late endosome of HeLa cells labeled with the anti‐LBPA monoclonal antibody 6C4, followed by 10 nm protein A‐gold (arrows). Bar: 0.1 μm. [Courtesy of Robert G. Parton, Brisbane, Australia]

LBPA seems to be ubiquitously distributed in all mammalian cells and tissues of high eukaryotic cells. However, with the possible exception of Dictyostelium,^41^ the lipid has not been detected in lower eukaryotes, including yeast. Prokaryotes^42^ and perhaps plants,^43^ however, contain the related lipid, acyl‐PG. Immunofluorescence and immunogold labeling of cryosections using a monoclonal antibody against LBPA revealed that the lipid is present exclusively in multivesicular regions of late endosomes and abundant in intralumenal membranes (Figure 3), a distribution further confirmed by subcellular fractionation.^44^, ^45^, ^46^ This distribution is consistent with the original finding—before endosomes had been characterized^47^—that LBPA is present in lysosomes.^39^, ^40^ While LBPA is a minor cellular lipid, it is abundant in these late endocytic compartments (late endosomes or endo‐lysosomes), where it may account for 15‐20 mol% of total phospholipids.^39^, ^40^, ^44^ This distribution is unique, because other phospholipids, in contrast to phosphoinositides,^48^ are not restricted to a subset of membranes of endocytic and secretory organelles, even though their relative abundance varies between organelles or membrane domains.^49^, ^50^, ^51^

### 2.1 Stereo‐configuration and biosynthesis

LBPA is an unconventional phospholipid not only because of its restricted distribution, but also because it exhibits a unique *sn‐1*‐glycerophosphate‐*sn‐1′*‐glycerol (*sn‐1:sn‐1′*) stereo‐configuration^52^, ^53^, ^54^ (Figure 2). LBPA is thus a poor substrate for most phospholipases,^46^, ^55^ and a perfect candidate to reside in the degradative environment of late endocytic compartments. However, despite its unusual headgroup and acyl chain organization, LBPA does not act like a detergent and has properties similar to other phospholipids.^56^

Both the unconventional stereo‐configuration and sub‐cellular distribution raise the issue of LBPA metabolism. In contrast to other phospholipids of the vacuolar apparatus that are synthesized in the early secretory pathway, LBPA is believed to be synthesized in late endocytic compartments from a phospholipid precursor. In vitro^57^ and in vivo^58^ observations have led to the notion that LBPA may be synthesized from phosphatidyl glycerol (PG), and that *sn‐3*‐PG could be converted into *sn‐1:sn‐1′*‐LBPA through a complex series of enzymatic reactions.^59^, ^60^ Since then, it has been shown that PG, but not cardiolipin, is indeed an LBPA precursor, but the biosynthetic pathway remains unclear.^61^ PG is synthesized in and confined to mitochondria—like cardiolipin that is synthesized from PG.^49^, ^62^ This raises the interesting possibility that mitochondrial PG may become available as LBPA precursor in late endocytic compartments through mitophagy.^63^

### 2.2 Trans‐bilayer distribution

The enzymes that mediate LBPA biosynthesis—or conversion from PG—should be present in the endosome lumen, a situation that may contribute to explain the restricted distribution of LBPA to late endocytic compartments. Newly synthesized LBPA is thus expected to be asymmetrically inserted into the exoplasmic leaflet of late endosomal membranes, including presumably ILV and limiting membranes. The presence of LBPA in the exoplasmic leaflet of the bilayer is consistent with observations that it binds endocytosed function‐blocking anti‐LBPA antibodies.^44^, ^64^, ^65^, ^66^, ^67^, ^68^, ^69^, ^70^, ^71^ Similarly, LBPA‐rich membranes may also serve as antigen for endocytosed antibodies associated with the antiphospholipid syndrome^44^, ^65^, ^70^ perhaps via beta(2)‐glycoprotein 1.^72^, ^73^

While LBPA is present in the exoplasmic leaflet of the bilayer, translocation across the bilayer to the cytoplasmic leaflet must occur because the lipid also interacts with the cytosolic ESCRT‐protein ALIX.^74^, ^75^ So far, no LBPA flippase has been identified. However, like other negatively‐charged phospholipids, LBPA may rapidly flip across the membrane if the charge were neutralized at low pH.^76^ The close proximity of the headgroups because of LBPA self‐assembly or clustering^46^ may cause partial protonation of proximal LBPA phosphate groups and transbilayer redistribution of the protonated form.^77^, ^78^ In turn, this may drive membrane shape changes, consistent with the capacity of LBPA to deform the bilayer in a pH‐dependent fashion^74^—keeping in mind that the redistribution of a very small fraction of phospholipids (< 0.1%) can induce significant shape changes.^78^ The unique features of LBPA are also illustrated by observations that, at the acidic late endosome pH, LBPA promotes liposome and virus fusion in vitro.^46^, ^79^ LBPA is thus present in both leaflets of the bilayer and on both ILVs and limiting membranes (Figure 3)—albeit more abundant in intralumenal membranes—and yet it is restricted to the multivesicular regions of late endosomes. Presumably, LBPA is preferentially incorporated into forming ILVs and may in fact play a direct role in ILV biogenesis^74^ (see also below), preventing LBPA redistribution to other membranes and ensuring replenishment of the lumenal content.

### 2.3 Acyl chain composition

In several cell‐types, LBPA is predominantly present as dioleoyl isoform (50%‐80%),^46^, ^80^ but the acyl chain composition of LBPA in rat liver and brain is more complex, including long polyunsaturated acyl chains.^81^ In vivo, acyl chains are predominantly present on the 2 and 2′ positions of the LBPA glycerol backbone, but these positions are unstable and the acyl chains can migrate to the 3,3′ positions^46^, ^82^ (Figure 2). It is not known to what extent acyl chain remodeling occurs in vivo and may accompany changes occurring in the intralumenal membrane organization. However, given the fact that the structures of these isomers are significantly different^83^ (Figure 2), it is likely that, in addition to the composition, the position of the acyl chains on the glycerol backbone determine LBPA shape and functions, and thus endosomal membrane dynamics. In fact, the peculiar structure of LBPA combined with its organization in LBPA‐rich membrane domains likely explain LBPA antigenicity.^44^, ^65^, ^70^

## 3 LBPA‐CONTAINING MEMBRANES CONTROL ENDOSOMAL LIPIDS

LBPA‐membrane play a crucial role in controlling the fate of other lipids, in particular sphingolipids and cholesterol, which are functionally linked in health^84^ and in sphingolipid and cholesterol storage disorders.^85^, ^86^, ^87^

### 3.1 Glycosphingolipid and ceramide degradation

Elegant biochemical studies have shown that LBPA‐rich membranes play a crucial role in the degradation of sphingolipids. This role has been discussed in comprehensive reviews,^88^, ^89^ and will only be briefly summarized below. In this process, sphingolipids are degraded in a stepwise manner by lysosomal enzymes with the help of saposins (Sap‐A, ‐B, ‐C, ‐D and GM2‐AP) in the presence of anionic phospholipids including LBPA.^90^, ^91^, ^92^ In vitro experiments showed that the degradation of the ganglioside GM2 can be stimulated 100‐fold by 20 mol% LBPA in the presence of GM2‐AP^93^—a concentration well in the range of LBPA levels in endosomes.^44^

### 3.2 Cholesterol transport

LBPA‐rich membranes also play a crucial role in controlling the fate of endosomal cholesterol. Most cells acquire cholesterol from circulating LDL endocytosed by the LDL receptor.^94^ Once in late endosomes, cholesteryl esters are de‐esterified and free cholesterol is rapidly incorporated into nearby membranes,^95^ including LBPA‐containing membrane. Cholesterol then reaches the endosome limiting membrane and becomes available for further export to the endoplasmic reticulum for cholesterol‐sensing,^96^ and to other organelles including the plasma membrane.^50^, ^97^ LBPA‐membranes also regulate the flux of cholesterol through endosomes during lipid droplet biogenesis induced by Wnt.^98^, ^99^ Cholesterol transfer from endosomes to the endoplasmic reticulum may be direct^12^, ^13^ or indirect via the plasma membrane,^100^, ^101^ and likely involves nonvesicular transport routes at membrane contact sites.^12^, ^13^, ^50^, ^95^

Within endosomes, cholesterol transfer to the limiting membrane depends on the proteins Niemann‐Pick type C1 and C2, and loss‐of‐function mutations in either of these proteins result in a cholesterol storage disease.^102^, ^103^ NPC1 is a multi‐spanning protein of the limiting membrane and NPC2 a globular protein present in the lumen,^104^, ^105^ and both proteins bind cholesterol.^106^, ^107^ Structural and mutagenesis evidence indicate that cholesterol is transferred from NPC2 to NPC1, thereby facilitating export from endosomes,^108^, ^109^, ^110^, ^111^, ^112^, ^113^, ^114^ and atomistic simulations indicate that LBPA is required for NPC2‐membrane interactions.^115^ Recent studies showed that NPC2 interacts directly with LBPA and that these interactions are necessary for cholesterol trafficking from endo‐lysosomes.^116^, ^117^ In addition, endocytosed antibodies against LBPA phenocopy NPC at the cellular level.^64^, ^66^, ^118^ Conversely, knockdown of the LBPA partner ALIX^74^, ^75^ decreases LBPA levels and endosomal cholesterol, suggesting that LBPA becomes limiting in NPC cells.^119^ Consistent with this view, a high‐content drug screen revealed that the small compound thioperamide raises LBPA levels, without affecting other endosomal functions, and concomitantly reduces the cholesterol overload in cells from Niemann‐Pick type C patients and in *Npc1−/−* mice.^81^ This compound is an inverse agonist of the histamine receptors H3/H4 and accordingly LBPA levels are inversely correlated with histamine receptor expression levels, but it is not known how this receptor controls LBPA levels.^81^ LBPA‐membranes may thus serve as platform to accommodate endosomal cholesterol, controlling both the cholesterol storage capacity of late endosomes and the flux of cholesterol through these organelles.

### 3.3 LBPA in NPC cells

Elevated levels of LBPA have been found in NPC^38^ and other lysosomal storage diseases.^120^, ^121^, ^122^ This increase may reflect some specific need for LBPA, for example in sphingolipid degradation.^89^ Alternatively, this increase may reflect the general expansion of the endo‐lysosomal compartment in storage disorders, upon upregulation of endo‐lysosomal gene expression by the transcription factor TFEB.^123^, ^124^ Consistent with the latter view, the increase in LBPA levels in NPC cells are correlated with the general expansion of late endosome volume, protein and lipid.^125^ Similarly, the elevated levels of LBPA in macrophages^80^ may reflect the higher degradative capacity of these cells.

Eventually, the cellular attempt to compensate for the accumulation of storage materials by an increase in the endosomal system collapses under the excess load in NPC cells and presumably in other storage disorders, leading to a traffic jam and a breakdown of endosomal membrane dynamics.^85^, ^86^ Given its role in endosomal cholesterol transport,^64^, ^98^, ^119^ LBPA may then become limiting^119^—and its capacity to accommodate or buffer excess cholesterol may be overwhelmed in NPC endosomes. Moreover, a lipidomic analysis revealed that, in addition to LBPA, the amounts of the LBPA‐related, minor lipid sLBPA (semi‐lysobisphosphatidic acid)^126^ (Figure 2) increases dramatically in the liver of *Npc1−/−* mice, up to the physiological levels of LBPA itself in WT mice.^81^ This analysis also revealed a profound and highly selective remodeling of the acyl chain composition of both LBPA and sLBPA in NPC mice, but not of any other phospholipid^81^—confirming the notion that a metabolic relationship exists between LBPA and sLBPA.^126^ One may thus speculate that such changes reflect some additional adjustment in LBPA‐membrane chemical and physical properties to better accommodate the changes caused by cholesterol accumulation.^127^, ^128^, ^129^

There is no approved treatment against NPC except for Miglustat, which delays but does not arrest the progression of the disease.^130^ Cyclodextrins clear cholesterol storage and restore cholesterol feedback regulation in NPC mice,^131^, ^132^, ^133^, ^134^, ^135^ improve symptoms and survival in NPC animal models,^136^, ^137^ and decrease the neurological progression of the disease in phase 1‐2 trials in NPC patients,^138^ suggesting that cyclodextrins may emerge as therapeutical strategy. However, the mechanism of action is being debated.^139^, ^140^ Recent studies indicate that hydroxypropyl‐cyclodextrin acts by promoting the secretion of the endo‐lysosome content, including LBPA, via a mechanism that requires the lysosomal cation channel mucolipin‐1 (MCOLN1 or TRPML1)^141^ (see Figure 6), which is itself responsible for the lysosome storage disease (LSD) mucolipidosis type 4 when mutated.^142^ Interestingly, endo‐lysosome secretion elicited by cyclodextrin in NPC cells decreases endosomal cholesterol but not total cell cholesterol, indicating that the secreted cholesterol is presumably incorporated into the plasma membrane or released and recaptured by cells, and eventually redistributed intracellularly.^141^ On the whole, these data fit nicely with observations that secretory endosomes or lysosomes^15^ mediate the secretion of storage material in lysosome storage disorders via activation of TFEB‐family transcription factors,^143^, ^144^, ^145^ and that the secretion of endo/lysosome storage materials depends on MCOLN1 activation.^146^, ^147^

## 4 BIOGENESIS OF INTRALUMENAL MEMBRANES

### 4.1 ILV and exosome biogenesis

Downregulated signaling receptors, and other proteins destined for late endosomes and lysosomes, are selectively sorted into ILVs, in a process that begins in early endosomes.^2^, ^148^ (Figure 1). Protein sorting into ILVs and ILV formation depend on endosomal sorting complexes required for transport (ESCRT)‐0, ‐I, ‐II and ‐III. In yeast an alternative intralumenal fragment pathway^149^ may also mediate the ESCRT‐independent downregulation of surface transporters delivered to the vacuole limiting membrane.^150^

**Figure 4:**
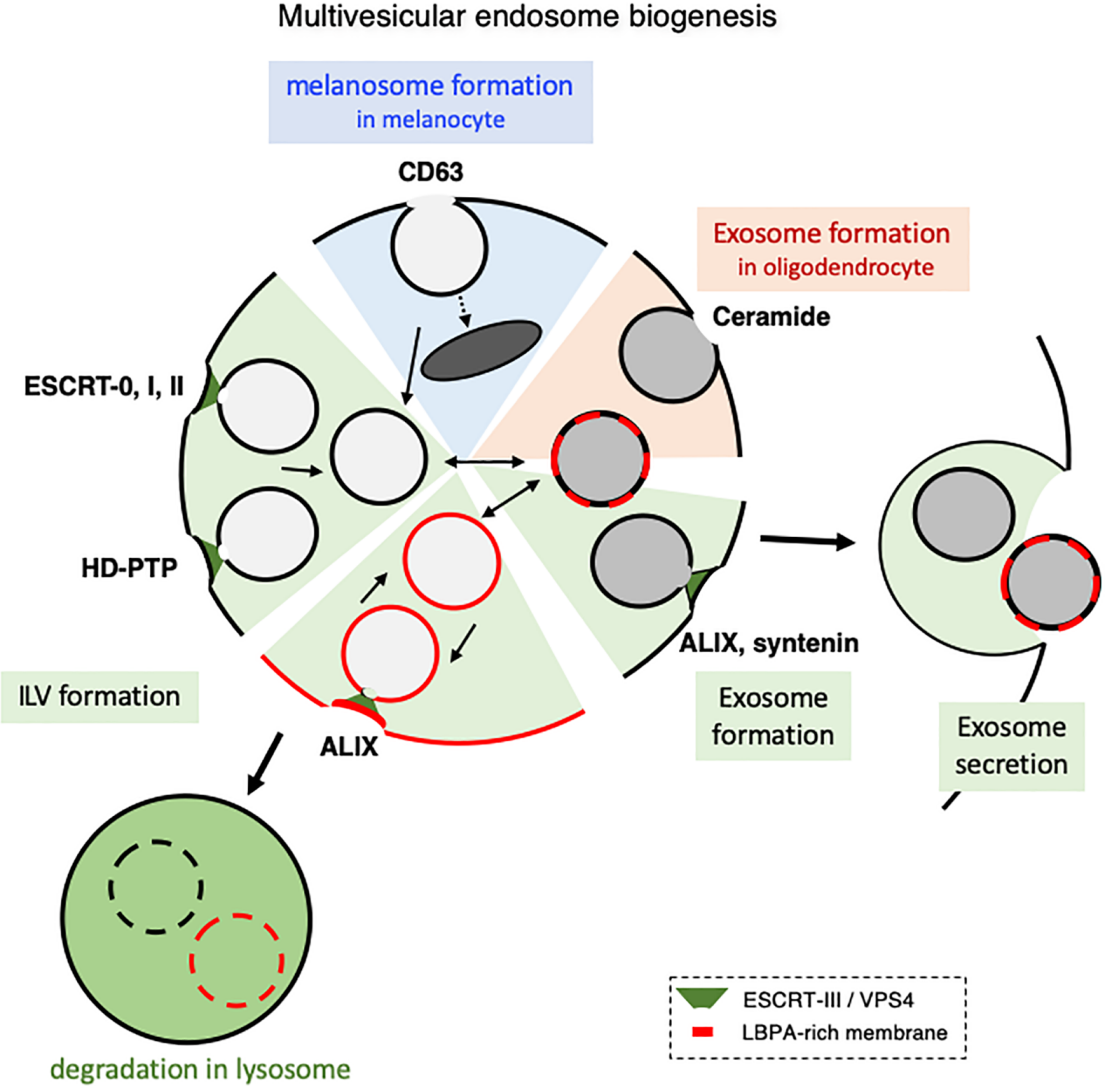
Multivesicular endosome biogenesis. The figure outlines the proposed mechanisms driving the formation of ILVs and exosomes in most cell types (green), exosomes in oligodendrocytes (brown) and melanosomes in melanocytes (blue). In most cell types, sorting into ILVs is mediated by ESCRT‐0, ‐I and ‐II, HD‐PTP or ALIX, as is presumably the nucleation of ESCRT‐III filaments, which drive the membrane deformation process. However, ILVs may also be formed in a CD63‐dependent and ESCRT‐independent manner—a process presumably akin to the biogenesis of melanosomes in melanocytes. ILVs formed in early endosomes presumably lack LBPA, because the lipid is only found in late endosomes. The biogenesis of exosomes may require ALIX and ESCRTs, as well as syntenin presumably, but not in oligodendrocytes where the process seems to depend on ceramides and to be ALIX‐ and ESCRT‐independent. Once formed, ILVs and exosomes follow different pathways. ILVs can be targeted to lysosomes for degradation, or undergo back‐fusion with the limiting membrane. Exosomes by contrast are secreted upon endosome fusion with the plasma membrane. The relationship between ILVs and exosomes are not clear. Neither are the mechanisms that discriminate their selective fates. The factors that have been reported to control each process are indicated. Membranes shown in the black color imply that it is not known whether the corresponding processes involve LBPA‐containing membranes

The current view is that ESCRT‐0 initiates the process by binding both PtdIns3P on the membrane and ubiquitin conjugated to cargo molecules, and recruits ESCRT‐I, which in turn recruits ESCRT‐II as nucleator for ESCRT‐III filaments^151^, ^152^ (Figure 4). In addition to ESCRT‐0, ‐I and ‐II, the filaments of ESCRT‐III can also be nucleated by other factors, including the LBPA partner ALIX,^30^, ^153^, ^154^ and perhaps HD‐PTP, which shares a Bro‐1 domain with ALIX.^155^, ^156^, ^157^ ALIX mediates the ESCRT sorting of the GPCRs PAR1 and P2Y1,^158^, ^159^, ^160^ while HD‐PTP is required for the downregulation of the EGF receptor,^155^ PDGF receptor,^161^ α5β1 integrin,^162^ and virally ubiquitinated MHC class I.^156^

In vivo and in vitro observations show that ESCRT‐III filaments drive the membrane deformation process that leads to ILV formation,^163^, ^164^, ^165^ presumably in conjunction with the triple A ATPase VPS4.^166^, ^167^ LBPA itself may also play a direct role in this process.^74^ In addition, ESCRT‐III drives other membrane deformation processes that share the same topology, including cytokinetic abscission, viral budding, nuclear envelope reformation,^168^, ^169^, ^170^, ^171^ as well as plasma membrane 172, 173 and endo‐lysosome membrane repair.^153^, ^154^, ^174^ Hence, ESCRT‐III functions as the general membrane deformation and fission machinery with an orientation opposite to endocytosis, away from the cytoplasm.

In addition to ESCRT‐dependent mechanisms, ILVs may also form via ESCRT‐independent pathways.^175^ In melanocytes, the melanosomal protein PMEL is sorted into ILVs in an ESCRT‐independent^176^ but CD63‐dependent manner^177^ (Figure 4). Similarly, different ILV populations may be formed in a Hrs‐ or CD63‐dependent manner in HeLa cells.^178^ It should be noted that EGF, which triggers EGF receptor endocytosis and sorting into ILVs, also increases multivesicular endosomes biogenesis and ILV formation^179^ in an ESCRT‐dependent manner.^175^ However, the mechanism driving the increase in ILV formation is not known, perhaps dependent on annexin 1^179^ and SCAMP3.^180^ In addition, stress exposure triggers the ligand‐independent internalization of EGF receptor via a route that diverts from the canonical pathway and that depends on WASH and Tsg101‐ALIX, leading to EGF receptor accumulation in a subset of LBPA‐rich multivesicular endosomes.^181^

### 4.2 Microautophagy and exosome biogenesis

In a process clearly reminiscent of ILV biogenesis, cytosolic components can be engulfed within the lumen of nascent ILVs via microautophagy, and then delivered to lysosomes.^7^, ^182^ Microautophagy may be mediated via more than one pathway, dependent or not on autophagy‐related (ATG) genes. In budding yeast, the NPC orthologs, Ncr1p and Ncr2p, promote microautophagy presumably by increasing sterol in the vacuole limiting membrane.^183^ In fission yeast, Nbr1 was identified as autophagy receptor for the ESCRT‐dependent targeting of soluble cargos to the vacuole.^184^ Accumulating evidence support the notion that the ESCRT machinery is required for microautophagy.^185^, ^186^, ^187^, ^188^, ^189^, ^190^ In addition, evidence also suggests that proteins encoded by ATG genes have pleiotropic effects on exosome biogenesis and release.^9^ In particular, the ATG3‐ATG12 conjugate was reported to interact with ALIX in order to promote autophagy and exosome biogenesis.^191^

Exosomes correspond to a sub‐population of extracellular vesicles that originate from ILVs and are released outside cells upon endosome fusion with the plasma membrane 31, 192, 193 (Figure 1). Consistently, exosome biogenesis depends on ESCRT‐III,^194^ and ALIX^71^, ^195^, ^196^—although exosomes secreted by oligodendrocytes may form in a ceramide‐dependent but ALIX‐ and ESCRT‐independent manner^197^ (Figure 4). In addition, LBPA is present in exosomes^198^ and ALIX is considered as one of the best‐established exosome markers,^31^, ^199^, ^200^ which is surprising given the fact that ESCRTs remain cytosolic and are typically excluded from ILVs.^201^, ^202^

Essentially nothing is known about the mechanisms that control the alternative fates of ILVs—degradation in lysosomes, back‐fusion or secretion as exosomes. Neither is anything known about the principles responsible for the lysosomal targeting of ILV cargoes or retrieval to other destinations, including exosomes.

### 4.3 Biochemically‐distinct populations of ILVs

The sub‐cellular distribution of LBPA clearly demonstrates that biochemically‐distinct populations of ILVs co‐exist within endosomes. Indeed, the lipid cannot be detected in early endosomes,^44^ where ILV biogenesis begins.^148^ Neither is the lipid detected in canonical multivesicular endosomes/bodies, which serve as intermediate between early and late endosomes (Figure 1). LBPA is found, and thus likely synthesized, in late endosomes or endo‐lysosomes,^44^ which are filled with internal membranes of various origins, including exosomes in the making, ILVs destined for lysosomes, as well as remnants of organelles delivered by autophagy (see tomogram of late endosomes in Cos cells—Movie S1). LBPA itself seems to be enriched in one sub‐population of these intralumenal membranes.^46^ Consistent with this notion, PtdIns3P and LBPA localize to different ILV populations within endosomes.^203^

The notion than more than one population of ILVs co‐exist in endosomes^204^ is clearly further supported by observations that, in addition to ESCRT‐dependent mechanisms, ILVs may also form via ESCRT‐independent pathways, as discussed above. One of the future challenges will be to establish what are the overlapping vs unique mechanisms, dependent or not on ESCRT subunits or ESCRT‐associated proteins, which may drive the biogenesis of functionally‐distinct populations of ILVs, microautophagosomes or exosomes. Interestingly, disruption of the class III PI3‐kinase Vps34 in neurons, which is required for both autophagy and ILV formation, triggers the secretion of unique exosomes enriched for undigested lysosomal substrates, specific sphingolipids, and LBPA.^205^

### 4.4 ILVs hijacked by pathogens

**Figure 5:**
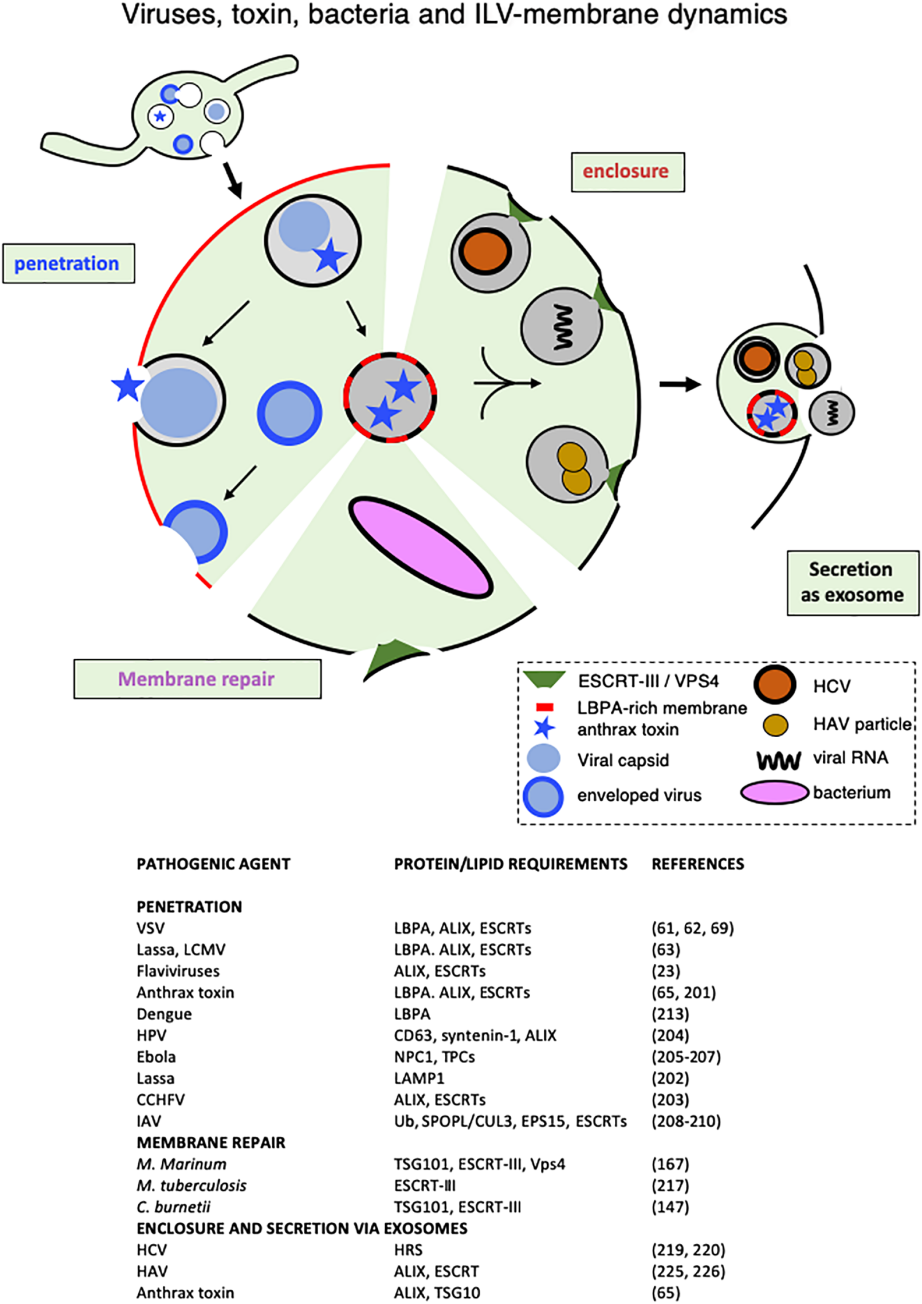
Viruses, toxin and ILV‐membrane dynamics. The left side of the figure (penetration) outlines the pathways used by some endocytosed pathogenic agents that enter the host‐cell cytoplasm through endosomes, in a process that depends on proteins/lipids involved in ILV membrane dynamics. VSV, Lassa virus, LCMV, and Flaviviruses may penetrate cells in a two‐step process. First, the viral enveloped undergoes fusion with the ILV membrane (eg, in early endosomes) so that the capsid be delivered into the protected environment of the ILV lumen. Then, the capsid is released into the host‐cell cytoplasm upon fusion of the ILV membrane with the late endosome limiting membrane (so‐called back‐fusion). Similarly, the anthrax toxin is first translocated across the ILV membrane and then delivered to the cytoplasm upon ILV back‐fusion. Other endocytosed viruses may penetrate cells upon direct fusion of the viral envelope with the late endosome membrane.^206^ The lower part of the figure outlines the role of ESCRT‐III and other ESCRT sub‐units in repairing damage to vacuoles containing the indicated bacteria. The right side of the figure outlines the inclusion of some viruses and viral particles into exosomes (enclosure) in a process that depends on ESCRT components, and their release as exosomes. The endocytosed anthrax toxin can also be released as exosomes, rather than being delivered to the cytoplasm of the target cell. Membranes shown in the black color imply that it is not known whether the corresponding processes involve LBPA‐containing membranes. CCHFV, Crimean‐Congo hemorrhagic fever virus; HAV, hepatitis A virus; HCV, hepatitis C virus; HPV, human papillomaviruses; IAV, influenza A virus; LCMV, lymphocytic choriomeningitis; VSV, vesicular stomatitis virus; *M marinum*, *Mycobacterium marinum* (in *Dictyostelium discoideum* cells); *M tuberculosis*, *Mycobacterium tuberculosis*); *C burnetii*, *Coxiella burnetii*

**Figure 6:**
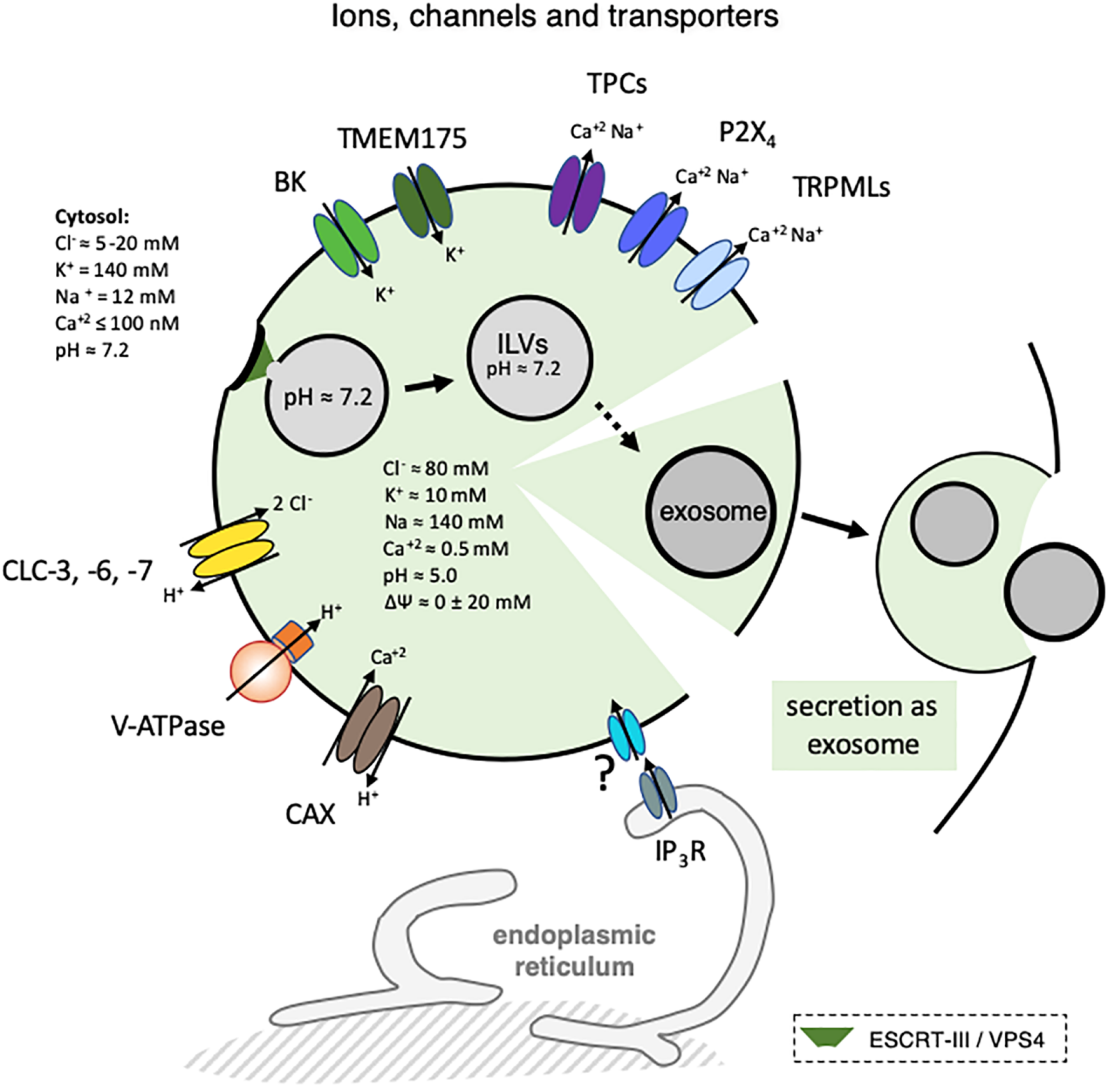
Ions, channels and transporters. The figure outlines the major ion channels and transporters present in endo‐lysosome, as well as the estimated ion concentration in the lumen of endo‐lysosomes and in the cytoplasm. The intralumenal concentration of Cl^−^ was estimated using a DNA‐based, fluorescent chloride reporter^271^ and see also.^272^ The lumenal concentration of Na is estimated to be around 140‐150 mM.^245^ Li and collaborators recently proposed that ΔΨ of resting lysosomes is around 0 (±20 mV).^235^ Essentially nothing is known about the ionic situation within ILVs or exosomes, except for the observation that ILVs remain neutral until at least 20 minutes after formation.^266^ At ER‐lysosome membrane contact sites, the ER may sequester lysosomal Ca^2+^,^273^ and ER Ca^2+^ may refill lysosomal Ca^2+^ stores.^274^ Ca^2+^ is released from ER stores via Ins(1,4,5)P_3_ receptor (IP_3_R) and calcium refilling of the endosomes may be driven by the proton gradient via a vertebrate Ca^2+^/H^+^ exchanger (CAX),^275^ or depend directly on the ER in a pH‐independent fashion.^276^ Membranes shown in the black color imply that it is not known whether the corresponding processes involve LBPA‐containing membranes. V‐ATPase: the vacuolar ATPase^240^; CLC‐3, ‐6, ‐7: the 2Cl^—^/H^+^‐exchangers CLC‐3, ‐6, ‐7 (chloride channels) that distribute in endo‐lysosomes^238^; CAX, a putative endo‐lysosomal Ca^2+^/H^+^ exchanger involved in Ca^2*^ uptake into endo‐lysosomes^275^; P2X_4_, purinergic P2X receptor subtype 4; TPC, two‐pore channels; TRPMLs, transient receptor potential channels; BK, big conductance Ca^2+^‐activated potassium channel^274^; TMEM175: K^+^‐selective channel^235^

Pathogens use all tricks in the book to overcome cellular defenses, and not surprisingly, they also exploit the multivesicular endosome pathway^206^ (Figure 5). The anthrax toxin penetrates the target cell in a process that depends on LBPA, ALIX and other ESCRTs.^71^, ^207^ Similarly, during vesicular stomatitis virus (VSV) infection, the release of viral RNA into the cytosol depends on LBPA, ALIX and ESCRTs,^67^, ^68^, ^75^ as do Lassa virus and lymphocytic choriomeningitis virus^69^—Lassa virus was also shown to depend on LAMP1.^208^ Crimean‐Congo hemorrhagic fever virus (CCHFV) infection may also depend on ALIX and ESCRTs,^209^ while Human Papillomavirus (HPV) infection may rely on CD63, syntenin‐1 and ALIX,^210^ and Ebola virus on NPC1 and cation two‐pore channels (TPC)^211^, ^212^, ^213^ (Figures 5 and 6). Influenza A virus (IAV) infection depends on VPS4, as well as ubiquitination,^214^ the SPOPL/Cullin‐3 ubiquitin ligase complex and its target EPS15.^215^, ^216^ Although the precise role of LBPA, ALIX and ESCRTs in infection or intoxication remains to be elucidated, it has been proposed that anthrax,^71^, ^207^ VSV^67^, ^68^ and Japanese encephalitis and yellow fever flaviviruses^217^ may hijack ILVs so that toxin or nucleic acid be delivered to the cytoplasm by ILV back‐fusion with the limiting membrane.^29^, ^30^, ^218^ Interestingly, in this context, the fusion of dengue virus^219^ and VSV 79, 220 during infection depends on anionic phospholipids including LBPA—as does the cytoplasmic entry of the non‐enveloped Bluetongue Virus capsid.^221^

The ESCRT machinery was also recently shown to play additional roles during bacterial infection, in light with a general role for ESCRTs in repairing endo‐lysosome membranes^153^, ^154^ and other membranes.^222^ Vacuoles containing the intracellular pathogen *Coxiella burnetii* recruit ESCRTs to maintain an intact vacuole, which presumably provides the bacterium with a replication advantage.^154^ Similarly, ESCRTs are required to repair small membrane damage in the vacuole containing *Mycobacterium marinum* in *Dictyostelium discoideum* 174 or *Mycobacterium tuberculosis* in macrophage,^223^ presumably to ensure that the pathogen remains contained within intact compartments.

ILVs as exosomes have also been proposed to mediate the spreading of pathogens or pathogenic agents from cell to cell (Figure 5). In fact, it is being discussed whether viruses and exosomes (or other types of extracellular vesicles) share similarities and may be related.^224^ It has been reported that exosomes may mediate the transmission of hepatitis C virus^225^ in a process that depends on the ESCRT subunit HRS.^226^ Similarly, exosomes have also been proposed to transfer hepatitis C viral RNA.^227^, ^228^ as well as nucleic acids from other viruses including HIV.^229^, ^230^ The non‐enveloped hepatitis A virus was also shown to be released after inclusion within a host‐derived exosomal‐like membrane generated in a process that depends on the ESCRTs, VPS4B and ALIX^231^, ^232^—an observation that blurs the classic distinction between enveloped and non‐enveloped viruses. In addition, uropathogenic *Escherichia coli* (UPEC), which targets lysosomes but avoids degradation by pH neutralization, can be expelled in exosomes by bladder epithelial cells, upon pH sensing via the calcium channel TRPML3 (TRP channel 3 or mucolipin 3)^233^ (see Figure 6). Finally, in addition to delivering their toxin cargo to the cytoplasm by back‐fusion, ILVs containing anthrax toxin may also be released as exosomes so that the toxin can be transmitted to naïve cells.^71^ Interestingly, however, anthrax toxin containing ILVs fail to be targeted to lysosomes for degradation.^71^ It thus appears that that the machinery controlling ILV formation and dynamics has been hijacked to mediate viral RNA or toxin release to the cytoplasm during infection/intoxication, or secretion to the extracellular medium as exosomes in order to propagate the infection or to spread the toxin to naïve cells.

## 5 LIFE IN THE LUMEN

### 5.1 Protons, anions and cations

In the late endosome lumen, where LBPA is found, ILVs and other intralumenal membranes are packed within a highly crowded environment (Movie S1). Beyond the diversity of membranes already discussed above, relatively little is known about the biochemical and biophysical properties of the lumenal milieu,^234^ although much progress has been made in the characterization of endo‐lysosomal ion channels and in the description of the ionic situation within the endo‐lysosomal milieu (for recent reviews, see^235^, ^236^, ^237^, ^238^, ^239^). It is well‐established that endo‐lysosome acidification depends on the V‐ATPase, with early endosomes having a mildly acidic pH ≈ 6.2 and late endosomes/lysosomes a more acidic pH ≈ 5.0^234^, ^240^, ^241^ (Figure 6). Numerous physiological processes, including ligand‐receptor uncoupling, lysosomal enzyme activity and membrane traffic are controlled by the acidification properties of endo‐lysosomes. The low endo‐lysosomal pH is also used by enveloped viruses to trigger fusion of the viral envelope with the endosomal membrane and by some toxins to cross the endo‐lysosomal membrane so that the viral nucleic acid or the toxin can reach the host‐cell cytoplasm.^206^, ^242^ In addition to protons, cations and anions also play important roles in the regulation of the endo‐lysosomal lumenal environment. Chloride controls ion homeostasis and endo‐lysosome acidification, and is regulated in endo‐lysosomes by 2Cl^−^/H^+^‐exchangers of the CLC anion transporter family (ClC‐3 through ClC‐7), which are responsible for several disorders when mutated^238^ (Figure 6).

In mammalian cells, endo‐lysosomes, in addition to the ER and mitochondria, also serve calcium storage functions—referred to as acidic calcium stores—presumably regulated via ER‐endosome membrane contact sites 12, 237, 243, 244 (Figure 6). Cation channels, including in particular the mucolipin subfamily of TRPML (transient receptor potential) channels or the distantly related TPCs (two‐pore channels) maintain endosomal calcium homeostasis,^237^, ^243^, ^244^, ^245^ and may also function as key regulators of endo‐lysosomal trafficking and autophagy‐related processes.^246^, ^247^ Calcium is indeed believed to play an important role in the regulation of endo‐lysosome and autophagosome membrane dynamics.^5^, ^246^, ^247^, ^248^ As already mentioned, mutations in TRPML1 (or mucolipin‐1, MCOLN1) are responsible for the LSD mucolipidosis type 4,^142^ and dysfunction of endo‐lysosomal calcium is observed in various LSDs.^249^, ^250^, ^251^ In addition, TPCs are involved in Ebola virus penetration from endo‐lysomes into host‐cells,^211^ while delivery of the viral core to the cytoplasm depends on the NPC1 protein 212, 213 (Figure 5). Finally, efflux of calcium from damaged endosomes serves as a signal to trigger an ESCRT‐mediated repair process.^153^

Much like in the ER,^252^ the free Ca^+2^ in endosomes is estimated to 0.4‐0.6 mM.^249^, ^253^ In the ER, most Ca^+2^ is buffered by abundant lumenal Ca^+2^‐binding proteins. 254, 255 However, these proteins or their functional homologs are not found in endosomes and lysosomes, and the nature of the Ca^+2^‐binding molecules that play similar roles in the acidic calcium stores is unknown. Yet, it can be estimated that ≈99.9% of Ca^+2^ in acidic stores is chelated, supporting the notion that buffer molecules or matrix must exist.^256^ It is appealing to propose that the abundant, negatively‐charged lipid LBPA serves as calcium buffer in the lumen of late endosome/endo‐lysosomes. Indeed, the capacity of calcium to bind negatively‐charged lipids is a universal principle, which is best illustrated by the active translocation of the negatively‐charged lipid PS from the outer leaflet of the plasma membrane (high calcium environment of the blood) to the inner leaflet (low calcium environment of the cell).^257^ Moreover, calcium exhibits a substantial capacity to bind membrane phospholipids^258^, ^259^, ^260^, ^261^ and to alter the properties of the bilayer.^262^ In fact, accumulation of the divalent cation Zn^+2^ in the LBPA‐containing late endosomes of cells expressing the ZnT2 zinc transporter caused cholesterol accumulation much like in NPC cells.^64^ It can be anticipated that calcium association to LBPA‐rich membranes in the endosome lumen may not only control the fate and dynamics of ILVs, but may also play a key‐role in the late endosome/endo‐lysosome capacity to modulate calcium‐dependent processes, including in lysosomal signaling.^246^

### 5.2 The lumen in the lumen: Size matters

In mammalian cells, typical ILVs form one or more fairly homogenous populations of vesicles with a mean diameter around 50 nm,^263^, ^264^ while ILVs in yeast are smaller with a diameter of ≈ 25 nm.^265^ Essentially nothing is known about the chemical conditions that exist within the lumen of ILVs and exosomes, beyond the observations that the pH of newly‐formed ILVs is neutral.^266^ One should keep in mind that the volume of a 50 nm diameter ILV is exceedingly small, corresponding to ≈ 65 × 10^−3^ aL, implying that a fraction of a proton only suffices to reduce the pH by two units, from 7 to 5. Whatever the fate of ILVs, degradation, secretion or retrieval, one may consider these vesicles as unit containers packaging quantum amounts of cargo in the membrane or in the lumen.

This notion becomes important when considering some of the ILV or exosome functions. For example, exosomes presumably transport miRNAs from donor to acceptor cells,^20^, ^21^, ^267^, ^268^ and thus regulate gene expression in target cells, by repressing translation of target mRNAs and/or by inducing their degradation.^269^ One miRNA targets a single RNA molecule, in contrast to enzymes that are regenerated during the catalytic cycle and can process many substrates. Thus, if incorporation into exosome was strictly passive, one would need 2‐16 × 10^6^ exosomes of 50‐100 nm diameter to transfer one miRNA species from one typical donor cell with a volume ≈ 1000 fL,^270^ to a target cell of the same volume in order to achieve the same miRNA concentration as in the donor—irrespective of what the concentration is—hence, a volume equivalent to the total volume of the donor cell. Thus, a highly efficient mechanism must exist to produce, sort and package miRNAs into exosomes, and to target these exosomes to the recipient cells, for such a transfer mechanism to operate in a physiologically‐relevant manner—miRNAs and RNAs associated to extracellular vesicles are reported to be enriched in certain sorting motifs.^224^

Using an assay that measures the biogenesis of ILVs into late endosomes in vitro, the ILV lumenal pH was found to be neutral for a relatively long time, up to 20 minutes after ILV formation.^266^ However, given the fact that an ATP‐dependent mechanism is unlikely to maintain the pH gradient across the ILV membrane inside endosomes, it is not known whether the pH gradient persists until digestion in the lysosomes, or whether proton permeation across the bilayer eventually acidify the lumen, prior to degradation. In any case, the asymmetry across the ILV membrane driven by pH and ion gradients, as well as the asymmetric protein and lipid composition of the ILV bilayer likely contribute to regulate the fate of ILVs.

## 6 CONCLUSION

Late endosomes/endo‐lysosomes are unique organelles of the vertebrate vacuolar apparatus in that they contain membrane vesicles within their lumenal environment, which is topologically equivalent to the extracellular space. These vesicles are highly specialized, in particular because some are rich in LBPA—an atypical lipid that is not found elsewhere in the cell. LBPA not only has an unconventional biosynthetic pathway and stereochemistry, but also has a unique shape and acyl chain migration capacity, likely to influence its impact on membrane organization and dynamics.

A fully unanswered and outstanding question is the nature of the mechanism that drive the sorting of ILVs toward one of their possible fates—degradation in lysosomes, secretion as exosomes, or recycling to the limiting membrane via back‐fusion. The privileged and secluded environment of ILVs, bathed into the late endosome/endo‐lysosome lumen, is fully disconnected from all cytosolic machineries that drive signaling or protein and lipid sorting, and therefore the fate of ILVs cannot rely on these established mechanisms. Future work will be needed to address this issue. However, some speculations are already possible. LBPA‐rich membranes are involved in the regulation of several features of the endo‐lysosome intralumenal membrane system, including cholesterol transport, sphingolipid degradation, and membrane dynamics, as well as perhaps endosomal Ca^+2^. LBPA also exhibits a rare capacity for adaptive shape changes, via acyl chain remodeling, because of its unique structure. It is therefore attractive to believe that LBPA‐rich membranes play a crucial role in modulating trafficking within the endosome and the fate and dynamics of intralumenal membranes. In particular, given the fact that the LBPA partner ALIX is involved in the biogenesis of at least some exosome populations and is itself found in exosome, LBPA‐rich endosomal membrane domains may ultimately control the biogenesis of exosomes.

## Supporting information


**Supplementary Movie S1** The Movie shows the tomographic reconstruction of a late endosome/endolysosome in Cos cells, which illustrates the complexity of the intralumenal membrane system, including multilamellar regions with a Russian Doll‐like organization. In this tomogram, ILVs are clearly visible (and definitely bilayered), as well as other intralumenal membranes, including presumably remnants of organelles delivered by autophagy. Cells were fast frozen, and then processed for rapid freeze substitution. Images represent a 300 nm section of a typical endosome. [Courtesy of Robert G. Parton, Brisbane, Australia]
